# Network Analysis Identifies Drug Targets and Small Molecules to Modulate Apoptosis Resistant Cancers

**DOI:** 10.3390/cancers13040851

**Published:** 2021-02-18

**Authors:** Samreen Fathima, Swati Sinha, Sainitin Donakonda

**Affiliations:** 1 Department of Biotechnology, Faculty of Life and Allied Health Sciences, MS Ramaiah University of Applied Sciences, Bengaluru 560054, India; samreenzfathima@gmail.com; 2 School of Medicine, Institute of Molecular Immunology and Experimental Oncology, Klinikum Rechts Der Isar, Technical University of Munich, 81675 Munich, Germany

**Keywords:** apoptosis resistant cancers, cell death, transcriptome, protein–protein interaction networks

## Abstract

**Simple Summary:**

With the thriving efficacy of cancers to evolve and evade treatment strategies targeting the apoptosis cell death mechanism, it has become imperative to reorient these conventional cancer therapy methods. In this study, we opted for an in-silico approach to mine the essential non-apoptotic cell death genes. We holistically examined the extensive protein–protein interaction networks in three such cancers with poor prognosis: colon adenocarcinoma, glioblastoma multiforme, and small cell lung cancer. Our analysis identified non-apoptotic cell death drug targets.

**Abstract:**

Programed cell death or apoptosis fails to induce cell death in many recalcitrant cancers. Thus, there is an emerging need to activate the alternate cell death pathways in such cancers. In this study, we analyzed the apoptosis-resistant colon adenocarcinoma, glioblastoma multiforme, and small cell lung cancers transcriptome profiles. We extracted clusters of non-apoptotic cell death genes from each cancer to understand functional networks affected by these genes and their role in the induction of cell death when apoptosis fails. We identified transcription factors regulating cell death genes and protein–protein interaction networks to understand their role in regulating cell death mechanisms. Topological analysis of networks yielded FANCD2 (ferroptosis, negative regulator, down), NCOA4 (ferroptosis, up), IKBKB (alkaliptosis, down), and RHOA (entotic cell death, down) as potential drug targets in colon adenocarcinoma, glioblastoma multiforme, small cell lung cancer phenotypes respectively. We also assessed the miRNA association with the drug targets. We identified tumor growth-related interacting partners based on the pathway information of drug-target interaction networks. The protein–protein interaction binding site between the drug targets and their interacting proteins provided an opportunity to identify small molecules that can modulate the activity of functional cell death interactions in each cancer. Overall, our systematic screening of non-apoptotic cell death-related genes uncovered targets helpful for cancer therapy.

## 1. Introduction

The apoptosis pathway is generally assigned as the only form of programed cell death that accounts for development and tissue homeostasis. However, animal models deficient of the necessary apoptotic machinery are known to develop naturally and attain adulthood [1,2]. So, cell machinery that is an alternative to the apoptotic methods of cell death is not only present but is capable of eliminating cells in the absence or malfunction of the apoptotic machinery. Apoptosis, immune cancer cells, are a significant roadblock in the mechanism of cell death in response to radiation and chemotherapy [3]. Due to the cancer cells’ malleable nature, the traditional chemotherapy and radiotherapy fall short, and a population of recalcitrant cancer cells begins to establish itself and cause recurrence. Cancers such as colon adenocarcinoma (COAD) [4], glioblastoma multiforme (GBM) [5] and small cell lung cancer (SCLC) [6] become resistant to conventional apoptosis, and available cancer therapies are rapidly proving inefficient in treating these types of cancers. Therefore, it is essential to find alternative mechanisms to induce cell death in these cancers.

As discussed, activation of alternative methods presents a lucrative modality. These may either support conventional apoptosis or occur independently of it. For example, caspase-3/8 inhibition can trigger necroptosis [7,8]. A better understanding of the relationship between necroptosis and cancer may help us to develop novel strategies for cancer therapy [9]. Pyroptosis is a caspase-1/4/5/11 dependent pathway that promotes inflammatory cell death and inhibits proliferation and migration of cancer cells [10]. Ferroptosis is morphologically, biochemically, and genetically distinct from other cell death forms [11]. Recently, other forms of cell death pathways such as alkaliptosis [12], entotic cell death [13], oxeiptosis [14], lysosome mediated cell death [15] and parthanatos [16] have been investigated as cancer therapeutics.

Complex diseases such as cancer involve perturbations of multiple biological pathways causing molecular alterations in the process of cell death. Hence, it is essential to comprehensively check the potential cell death pathways in the resistant cancer cells. To accurately distinguish the forms of cell death, we need to perform functional tests such as in silico, in vitro, and in vivo studies. We recognize these complex pathways operate through a highly organized protein–protein interaction network (PPIN) to produce a functional output [17]. PPIN allows us to systematically dissect the essential proteins connected with various functional proteins that ultimately lead to pathway readout [18]. Recent reports suggest that we can explore protein–protein interaction binding sites to modulate the functionality of the two interacting protein’s using drug molecules [19]. Thus, we can take protein–protein interactions (PPIs) can be taken further to predict the drug molecules that can modulate cell death pathways in cancer tissues. The development of novel drugs for selectively activating such pathways holds great promise for eliminating malignant cells.

In this study, we studied apoptosis-resistant cancers COAD, GBM, and SCLC and screened them for the non-apoptotic cell death genes (NACDGs) in their transcriptome profiles. Our systematic network-level analysis directed us to dissect distinct cell death signaling pathways and identify molecular effectors for evaluating pro-survival or reprograming mechanisms in apoptotic resistant cancers. Our analysis also allowed us to discover the probable therapeutic molecules that can open up possibilities of inducing non-conventional cell death in apoptotic immune cancer cells.

## 2. Results

### 2.1. In Silico Workflow to Identify the Non-Apoptotic Cell Death Genes in Cancers

Evasion of apoptosis, the molecular level pathway involved in cell death, is known to cause resistance to cancer therapy [20]. Reports suggest that COAD, GBM, and SCLC progressively become resistant to apoptosis mediated cell death, and are hence immune to conventional chemotherapy or radiotherapy [4,5,6]. This prompted us to dissect NACDGs in COAD, GBM, and SCLC.

**Figure 1:**
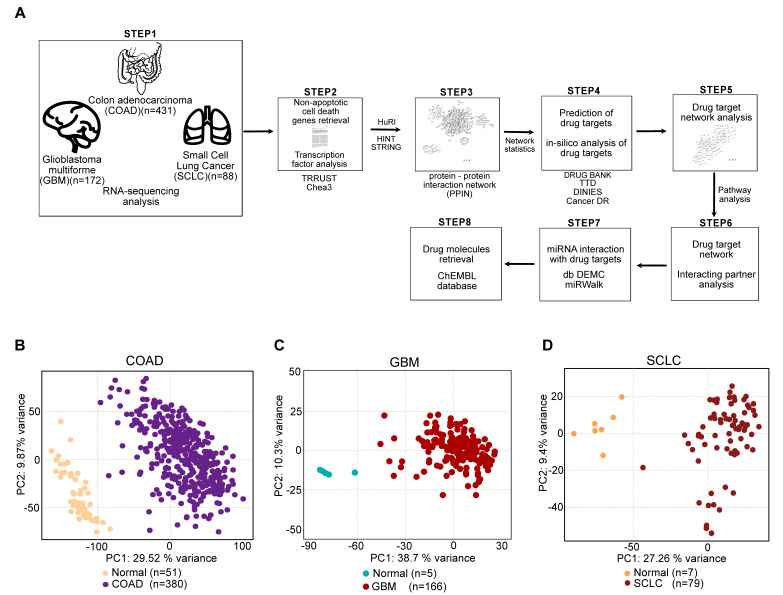
RNA-sequencing analysis of colon adenocarcinoma (COAD), glioblastoma multiforme (GBM) and small cell lung cancer (SCLC). (**A**) The schematic representation of data collection and integrative analysis. (**B**–**D**) Principal component analysis (PCA) built on RNA-sequencing datasets of COAD (PC1 = 29.52%, PC2 = 9.87%), GBM (PC1 = 38.7%, PC2 = 10.3%) and SCLC (PC1 = 27.26%, PC2 = 9.4%).

In this current study, we utilized a computational pipeline, as shown in Figure 1A. We extracted the gene expression datasets of COAD and GBM, of normal and cancer patients, from The Cancer Genome Atlas (TCGA). The SCLC gene expression dataset (normal and cancer patients) was extracted from Jiang L et al. [21]. We followed an identical statistical analysis approach to analyze the datasets and performed literature mining to extract NACDGs. Then we predicted the upstream transcription factors (TFs) and constructed the protein–protein interaction networks of NACDGs. Next, we performed the drug target prediction, miRNA drug target interaction and drug target network analysis. Finally, we screened for drug molecules against the drug targets, targeting their protein–protein interactions in each cancer phenotype.

### 2.2. Gene Expression Analysis of COAD, GBM, and SCLC and Regulation of Non-Apoptotic Cell Death Genes

To understand the governing effects in COAD, GBM, and SCLC, we separately analyzed gene expression profiles of these tumors. The principal component analysis (PCA) revealed clear segregation between normal and cancer patients (Figure 1B–D). We identified following number of differentially expressed genes (DEGs) in cancer datasets: 4788 (COAD, 3214 up, 1574 down) (Table S1), 6575 (GBM, 3444 up, 3131 down) (Table S2) and 4026 (SCLC, 2575 up, 1451 down) (Table S3).

**Figure 2:**
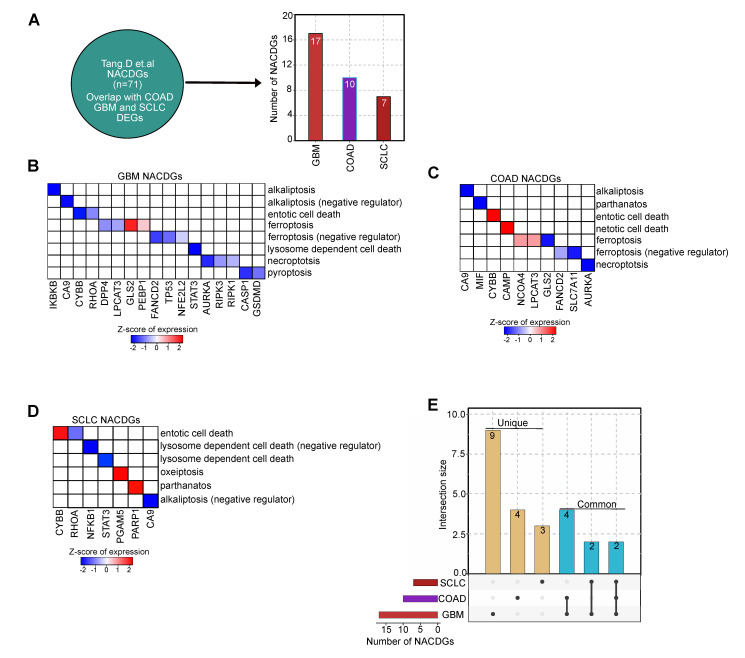
Literature evaluation of non-apoptotic cell death genes (NACDGs). (**A**) The overlap between NACDGs in each cancer dataset. (**B**–**D**) The heatmaps represents the regulation of NACDGs in COAD, GBM and SCLC. (**E**) The UpSet plot shows the unique (single black dot) and common (connecting black dots) NACDGs across SCLC, COAD and GBM.

Next, we investigated the DEGs in each cancer responsible for NACDGs by mining the literature (see methods) in GBM (*n* = 17), COAD (*n* = 10), and SCLC (*n* = 7) (Figure 2A). This analysis revealed the following in the GBM: we observed 2 upregulated and 15 downregulated NACDGs (Figure 2B, and Table S4a). On the other hand, in COAD, 4 and 6 NACDGs are up and down-regulated (Figure 2C and Table S4b). Furthermore, in SCLC, 3 and 4 are up and down-regulated (Figure 2D and Table S4c). Herein after, we focused our analysis only on NACDGs. We performed intersection analysis between NACDGs and found that CYBB and CA9 are shared among all cancer datasets. Whereas LPCAT3, AURKA, GLS2, and FANCD2 are shared between the COAD and GBM, and RHOA, STAT3 are present in GBM and SCLC (Figure 2E and Table S5). Overall, our transcriptome analysis identified a wide variety of non-apoptotic cell death-related genes involved in cell death in these three cancer phenotypes.

### 2.3. Upstream Transcriptional Regulation of Cell Death Genes in COAD, GBM, and SCLC

**Figure 3:**
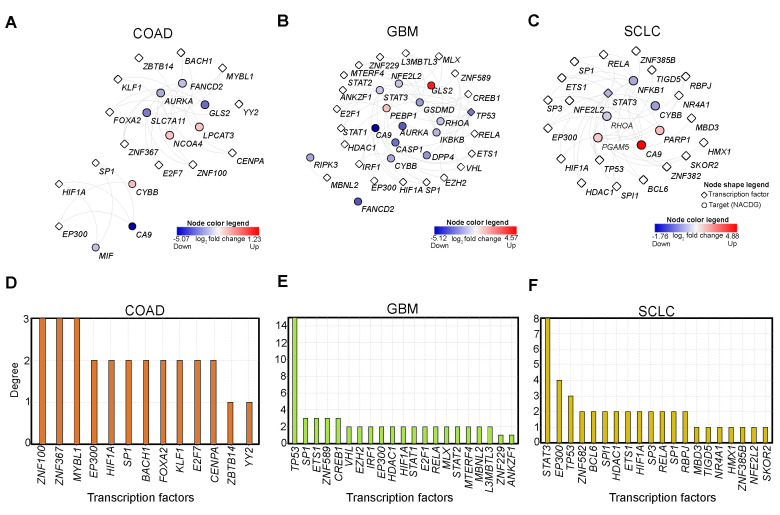
Transcription factor network analysis of NACDGs. (**A**–**C**) Network plots represent the predicted transcription factors (TF) regulating the NACDGs in COAD (*n* = 13 TFs), GBM (*n* = 21 TFs) and SCLC (*n* = 20 TFs). (**D**–**F**) Bar plots show the degree of each TF in TF-target networks. Note: red and blue color represent the up and down-regulation, respectively.

Transcription factors (TFs) are vital for regulating gene expression of various genes (targets), and transcriptional response is the central guiding force to all biological systems. Given their importance, we investigated the upstream TFs that regulate NACDGs in each cancer. To predict upstream TFs of NACDGs, we utilized TRRUST v2 and Chea3 tools [22,23]. This analysis identified 13 TFs, 21 TFs, and 20 TFs in COAD, GBM, and SCLC respectively (Figure 3A–C).

Next, we aimed to identify the prominent TFs based on the number of targets in each TF-target (TF-NACDGs) network. We computed degree of each TF in the network and found *ZNF100* (3 NACDGs) in COAD (Figure 3D). In the GBM TF-NACDG network, we found *TP53* (15 NACDGs) (Figure 3E). In SCLC, STAT3 (8 NACDGs) emerged as a critical TF (Figure 3F). In sum, our analysis identified key TFs which can regulate NACDGs in COAD, GBM, and SCLC, respectively.

### 2.4. Protein–Protein Interaction Networks Regulated by Cell Death Genes in COAD, GBM, and SCLC

To gain a deeper understanding between the NACDGs and their functional interacting proteins, connected with a variety of tumorigenic pathways in COAD, GBM, and SCLC, we analyzed PPIs between the protein products of the NACDGs and their interacting proteins (note: In the following sections, we will discuss them in the context of protein as NACDPs). We logged the NACDGs into the following databases to extract the PPIs: Human Reference Interactome map (HuRI), High-quality InTeractome database (HINT), and Search Tool for the Retrieval of Interacting Genes/Proteins (STRING DB). Through this approach, we generated networks consisting of 41 interactions between 56 proteins, 297 interactions among 794 proteins, and 147 interactions among 319 proteins in the COAD, GBM, and SCLC, respectively (Figure S1A–C). We further analyzed these NACDPs protein–protein interaction networks (NACDP-PPINs) to understand the topological structure. These analyses demonstrated that our PPINs follow a power-law distribution and scale-free property (R = 0.61, 0.81, and, 0.81) (Kolmogorov–Smirnov (KS) = 0.24, *p* = 0.87, COAD), (Kolmogorov–Smirnov (KS) = 0.18, *p* = 0.95, GBM) and (Kolmogorov–Smirnov (KS) = 0.18, *p* = 0.98, SCLC) Clauset test (Figure S2A–C).

Next, we ratiocinated the pathways enriched in the NACDP-PPINs in COAD, GBM, and SCLC datasets. These analyses revealed that NACDP-PPINs in COAD is involved in cell death and cell cycle pathways (Figure S3A). On the other hand, the NACDP-PPINs in the GBM were mainly enriched in cell death and signaling pathways (Figure S3B), whereas in SCLC, the NACDP-PPINs are involved in the signaling pathways such as MAPK, Rho, and NFκB pathways (Figure S3C). In sum, this analysis reinforced our workflow to illustrate that PPINs in each cancer are scale-free and play a role in a wide variety of tumor-related pathways.

### 2.5. Essential Proteins in Protein Interaction Networks Serve as Drug Targets in COAD, GBM, and SCLC

**Figure 4:**
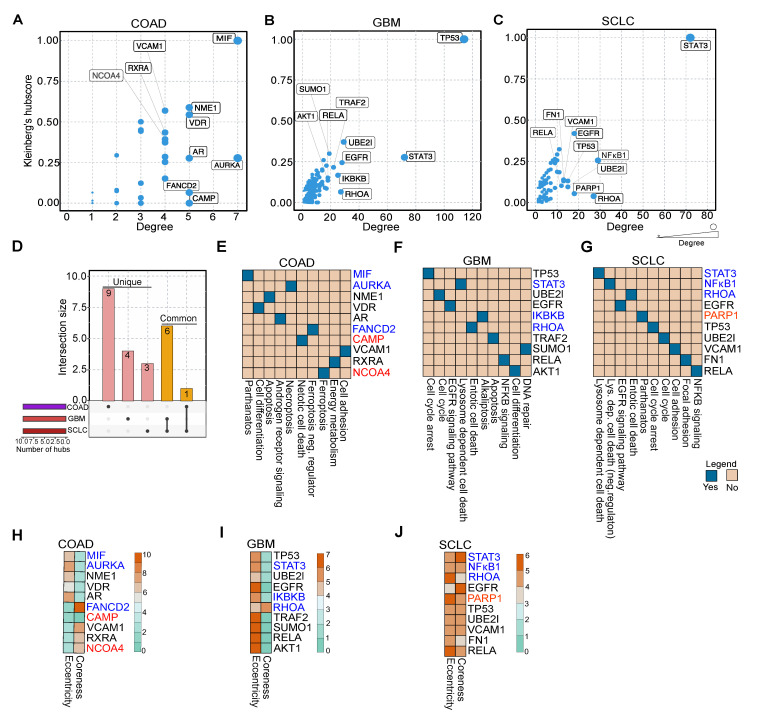
Hub protein analysis of NACDP protein–protein interaction networks. (A–C) The topology analysis of COAD, GBM, and SCLC protein–protein interaction networks, including Kleinberg’s hub centrality score and degree, divulge the top 10 hub proteins. (D) The UpSet plot shows the common and unique hubs across the cancer datasets. (E–G) Pathways of top 10 hub proteins in COAD, GBM, and SCLC. (H–J) Heatmaps represent the eccentricity and coreness of hub proteins in COAD, GBM, and SCLC, respectively. Note: Red and blue color denote up and down-regulation, respectively.

Earlier reports suggest that hub proteins in PPINs are essential for proper functional readouts [24]. To scrutinize which proteins in NACDP-PPINs (as described above) are hubs, we computed the degree and Kleinberg’s hub score of each protein in the PPINs to identify the highly connected proteins. We considered the top 10 proteins, which showed high degree and Kleinberg’s hub score for further analysis; this allowed us to identify the vital proteins in each cancer dataset. In COAD macrophage migration inhibitory factor (MIF, down), vitamin D receptor (VDR), NME/NM23 nucleoside diphosphate kinase 1(NME1), androgen receptor (AR), aurora kinase A (AURKA, down), vascular cell adhesion molecule 1 (VCAM1), retinoid X receptor alpha (RXRA), nuclear receptor coactivator 4 (NCOA4, up), FA complementation group D2 (FANCD2, down) and cathelicidin antimicrobial peptide (CAMP, up) (Figure 4A). In GBM tumor protein 53 (TP53), ubiquitin-conjugating enzyme E2L (UBE2l), signal transducer and activator of transcription 3 (STAT3, down), epidermal growth factor receptor (EGFR), inhibitor of nuclear factor-kappa B kinase subunit beta (IKBKB, down), ras homolog family member A (RHOA, down), AKT serine/threonine kinase 1 (AKT1), small ubiquitin-like modifier 1 (SUMO1), RELA proto-oncogene, NF-kB subunit (RELA), TNF receptor-associated factor 2 (TRAF2) identified as hubs (Figure 4B). On the other hand, in SCLC STAT3 (down), EGFR, TP53, nuclear factor kappa B subunit 1 (NFKB1, down), Poly (ADP-ribose) polymerase 1 (PARP1, up), RHOA (down), UBE2l, VCAM1, fibronectin 1 (FN1) and RELA were recognized as hubs (Figure 4C). To validate the hub molecules identified using the Kleinberg hub centrality hub score and degree, we computed two independent centrality parameters: closeness (an indicator of communication) and betweenness (an indicator of information flow). This analysis revealed 80% of hub proteins identified in Kleinberg’s hub score, and degree analysis (Figure S4A–D) also showed high closeness and betweenness, thus corroborating our hub analysis. We compared hubs to identify the common and unique hubs across the cancer datasets. This analysis revealed that VCAM1 is present in both COAD and SCLC, and RHOA (down), RELA, TP53, EGFR, UBE2l, STAT3 (down) are shared by the GBM and SCLC (Figure 4D and Table S6). The essential proteins in COAD are involved in cell death (NACDP) and differentiation pathways (Figure 4E). In GBM, the hubs play a role in cell cycle arrest, cell death (NACDP), and signaling pathways (Figure 4F)**,** whereas the hubs in SCLC are involved in cell death (NACDP) and EGFR signaling pathways (Figure 4G).

Apart from the hubs, we aimed to screen the NACDP-PPINs in each cancer dataset for drug targets. For this purpose, we calculated the following two scores: the first one is eccentricity, which shows the protein’s ability to be functionally available to all other proteins in NACDP-PPIN. The second one is coreness: this reveals the protein’s local and global importance in a given NACDP-PPIN. We utilized these measures to cull the drug targets in each cancer PPINs. As the main focus of our study is to identify critical non-apoptotic cell death genes, our examination uncovered the following NACDPs: MIF (down, pathanatos), AURKA (down, necroptosis), FANCD2 (down, ferroptosis (negative regulator)), CAMP (up, netotic cell death) and NCOA4 (up, ferroptosis) in COAD (Figure 4H), STAT3 (down, lysosome dependent cell death), IKBKB (down, alkaliptosis) and RHOA (down, entotic cell death) in GBM, and STAT3 (down, lysosome dependent cell death), NFκB1 (down, lysosome dependent cell death negative regulator), RHOA (down, entotic cell death) and PARP1 (up, pathanatos) in SCLC, respectively (Figure 4I–J). Interestingly, all these targets showed high eccentricity and coreness values; thus, we considered them as a drug target. Taken together, our topological analyses revealed essential proteins in PPINs and drug targets in each of the cancer dataset. 

### 2.6. Drug Target Validation and miRNA Regulatory Effect in COAD, GBM, and SCLC

**Figure 5:**
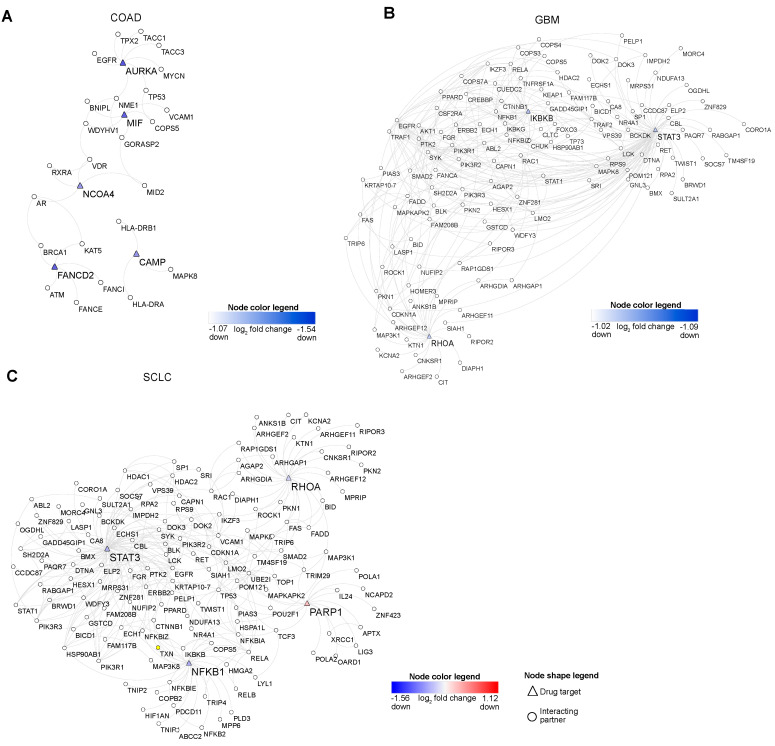
Drug target networks. (**A**–**C**) These network plots represent the drug targets and their interacting partners in COAD, GBM, and SCLC. Note: node color red and blue signify the up and down-regulation.

As discussed above, our NACDP-PPINs topology analysis forecasted the drug targets in COAD, GBM, and SCLC cancer datasets. We used these predicted drug targets to derive the subnetworks from PPINs (Figure 5A–C). Our pathway enrichment analysis of the COAD drug-target subnetwork showed that mitosis, Fanconi anemia pathway, and ER-stress are modulated (Figure S5A). The GBM drug-target subnetwork is enriched with the signaling pathways (MAPK and Rho), cell death, and cell motility (Figure S5B). On the other hand, the SCLC drug-target subnetwork is enriched with regulating cell death, MAPK, and NFκB signaling pathways (Figure S5C). To filter the key new drug targets, we further performed in silico validation of the predicted drug targets using various drug-target databases, such as the Drug Bank, Therapeutic Target Database (TTD), Drug-target Interaction Network Inference Engine Based on Supervised analysis (DINIES), and Cancer Drug Resistance Database (Cancer DR) (see methods). This analysis shows that in COAD, AURKA (down) is identified in all databases, MIF (down) and CAMP are present in 2/4 databases. Interestingly, we found two targets, FANCD2 (down) and NCOA4 (up), are not present in any of the database (Figure S6A). In GBM, STAT3 (down) is spotted in (3/4) databases and IKBKB, RHOA (1/4) databases (Figure S6B). In SCLC, NFκB1 (down) is acknowledged in all databases. PARP1 (up) and STAT3 (down) are found in (3/4) databases, whereas RHOA is identified only in one resource (Figure S6C). Finally, we considered targets which are not found in any or found in only one drug target database as a new valid drug target. Thereby, we proceeded our analysis by considering FANCD2, NCOA4 (COAD), IKBKB (GBM), and RHOA (GBM and SCLC) as the central targets. 

It is known that miRNAs are dynamically associated with NACDP-PPINs [24]. Therefore, we explored the possible miRNA regulatory effect on drug targets in COAD, GBM, and SCLC. We extracted miRNAs based on the prominent expression profile. This analysis enabled us to identify COAD specific 202, 204 miRNAs targeting FANCD2 and NCOA4, respectively (Figure S7A). Based on the expression, further investigations revealed that hsa-miR-492 (log_2_FC 1.37) miRNA is highly upregulated, targeting FANCD2 and NCOA4. In the GBM, we identified 98 miRNAs targeting IKBKB (Figure S7B). Dissection of these modules captured highly upregulated miRNA hsa-miR-138-5p (log_2_FC 8.11) which regulates IKBKB. Finally, in GBM and SCLC, we identified 73 miRNAs regulating RHOA. Further anatomization of these miRNA networks identified highly upregulated miRNA hsa-miR-181a-5p targeting RHOA (log_2_FC 5.32) (Figure S7B). Overall, in silico database analysis identified new drug targets, and our miRNA network analyses showed important cancer-specific miRNAs regulating drug targets.

### 2.7. Drug Target Network Predicts Key Interactions in COAD, GBM, and SCLC and Small Molecules

Next, we aimed to understand the critical functional interactions of those drug targets in each cancer. Towards this end, we selected each drug target’s interacting proteins of each drug target based on the vital tumor-related pathways in each tumor. In COAD, we found FANCI, which plays a role in Fanconi anemia pathway, as an interacting partner of FANCD2 (down, ferroptosis negative regulator). The second target in COAD, NCOA4 (ferroptosis, up), interacts with apoptotic protein RXRA. In GBM we identified KEAP1, a regulator of the apoptotic pathway, as an interacting partner of IKBKB (down, alkaliptosis). RHOA (down, entotic cell death) interacts with RHO signaling and apoptotic molecule ARHGEF2 in GBM and SCLC. 

Once we had these interaction data in hand, we sought to predict the partner-specific interface residues between the drug target and their interacting partner at the structural level. To do this, we retrieved experimentally derived structures of FANCD2, FA complementation group I (FANCI), RXRA, IKBKB, KElch-like ECH associated protein 1 (KEAP1), RHOA, and Rho/Rac guanine nucleotide exchange factor 2 (ARHGEF2) from the PDB database. However, we did not find the structure of the drug target NCOA4 protein structure; thus, we computationally modeled the 3D structure of NCOA4 using ITASSER (Figure S8A). We performed energy minimization and removed steric clashes followed by validation using ProSA by comparing the 3D structure of NCOA4 with experimental structures (X-ray and NMR). This analysis showed that the NCOA4 model is in the range of X-ray based structure (z-score: −5.98) (Figure S8B). We executed protein–protein docking using the HDOCK server (see methods) and identified critical residues of each drug target responsible for interaction with the above discussed interacting partners in cancer datasets (Figure S9A–C). 

To identify the drug molecules which can target the protein–protein interactions of predicted drug targets we extracted drug molecules from ChEMBL database for each target. This analysis highlighted Doxorubicin hydrochloride and Lenvatinib targets FANCD2, NCOA4 (COAD). Wortmannin targets IKBKB (GBM), and Clausine E binds to RHOA (GBM, SCLC). Overall, our drug-target interaction network analysis revealed vital drug targets and their key functional interacting partners along with drug molecules in COAD, GBM, and SCLC cancers. 

## 3. Discussion

The primary mechanism of cell death in response to chemo and radiotherapy is apoptosis. It is the failure of apoptosis in cancer cells that causes a severe clinical problem. Due to apoptosis pathway resistance, COAD, GBM, and SCLC hinder the treatment strategies in clinics. As an alternative route of cell death in these cancers, it is imperative to target non-apoptotic pathways for new anticancer drugs. So, activation of non-conventional methods of cell death holds the promise of cancer amelioration.

Recent advances in cell death mechanisms have brought the alternatives to apoptosis into focus [24]. The abundance of genomic data related to these cancers allows us to explore genes responsible for various non-conventional cell death pathways. In this study, we extracted normal and patient transcriptome profiles of COAD, GBM, and SCLC. Analysis of these datasets highlighted clusters of NACDGs involved in alkaliptosis, entotic cell death, ferroptosis, netotic cell death, necroptosis, lysosome dependent cell death, parthanatos and oxeiptosis pathways (Figure 2B–D). 

Parthanatos was previously linked to GBM [25], and our current research shows its involvement in COAD and SCLC. Mechanisms such as entotic cell death is observed in the breast [26], colon [27], and pancreatic carcinoma [28]. Non-apoptotic mechanisms involving autophagy proteins and lysosome-mediated cell digestion could also be potentially targeted in cancer tissues. In cancer cells that have an increased iron demand compared to non-cancer cells. This iron dependency can make cancer cells more vulnerable to iron-catalyzed necrosis, referred to as ferroptosis [29]. These observations suggest that alternative cell death pathways can be activated in apoptotic resistant cancers, and this offers promise in cancer therapy.

We used TRRUST and ChEA tools to identify the upstream transcription factors (TFs) responsible for the transcriptional response for each NACDGs in the cancer phenotypes. This approach revealed that 13 (COAD), 21 (GBM), and 20 (SCLC) TFs are possibly regulating NACDGs (Figure 3A–C). Further analysis of TF-target networks identified *ZNF100* (COAD); the factor known to sensitize the tumor cells [30], *TP53* (GBM), a well-known regulator of cell cycle arrest, and cell death pathways [31] and STAT3 (SCLC), which plays a role in cell proliferation, survival [32] (Figure 3D–F). TFs control a high number of NACDGs in these three recalcitrant cancers.

After identifying the transcription factors controlling the NACDGs, we investigated the proteins that interact with the NACDGs gene product, to gain further insight into their role in inducing cell death in these three cancers, individually. The NACDP-PPINs follow a scale-free pattern (Figure S2), and topological analysis exposed the top 10 hub proteins in COAD, GBM, and SCLC (Figure 4A–C and Figure S4). NACDP-PPINs play a role in various signaling pathways and cell growth-related functions. Furthermore, hub proteins were subjected to further analysis to predict drug targets (Figure 4G–I). We cross-validated these predicted drug targets with multiple drug-target databases (Figure S6A–C). 

We identified two drug targets in COAD as follows: the first one is FANCD2 (ferroptosis negative regulator, down), and it is involved in ferroptosis, a newly described cell death mechanism potentially used as cancer therapy [33]. The second one is NCOA4 (ferroptosis, up). The overexpression of NCOA4 is known to promote ferroptosis [34]. In GBM, we observed IKBKB (down, alkaliptosis) as a target; it regulates alkaliptosis in tumor tissues [12]. Intriguingly, we observed RHOA (down, entotic cell death) as a common drug target in GBM and SCLC. Entosis regulates the malignant cells [35]. We found the following TFs targeting drug targets: TF ZNF100 targets FANCD2 and BACH1, KLF1, ZBTB14 interacts with NCOA4 in COAD. TP53 TF interacts with IKBKB (GBM) and RHOA in (GBM and SCLC). This validates our presumption that TFs play a significant role in guiding protein interaction of the NACDGs. Further experimentation is needed to delineate the molecular mechanism of TFs targeting the drug targets in each cancer. 

miRNAs are known to regulate the protein production [36], and they also play an important role in harmonizing the protein abundance [37]. Thus, to interpret the post-transcriptional regulation on drug targets predicted from protein interaction analysis, we performed miRNA-protein interaction analysis. These analyses revealed that hsa-miR-492 is a significantly upregulated miRNA targeting the FANCD2, NCOA4 in COAD (Figure S7A). miR-492 is known to enhance tumor growth inhibition in colorectal cancer [38]. hsa-miR-138-5p (up) regulating IKBKB in GBM (Figure S7B), is reported to suppress tumor development in GBM [39]. We observed that hsa-miR-181a-5p (up) connected to RHOA in GBM and SCLC (Figure S7B) is known to regulate the cell growth pathways in tumors [40]. Thus, we believe that the connection between identified miRNAs and cell death related drug targets will open up therapeutic avenues in treating cancer.

Given our findings, we went on to analyze the drug-target interaction networks. Our pathway analysis augmented that these networks are involved in various pathways related to signaling pathways and tumor growth-related pathways (Figure 5 and Figure S5). In modern drug discovery, one of the preferred methods is to target PPIs [41] with drug molecules that can efficiently modulate the interactions responsible for multiple functions. This prompted us to explore how we can use drug targets and their interacting proteins for therapeutic purposes. To this end, we mined interacting partners of drug targets based on the functions important for tumorigenesis. This approach revealed the FANCI (Fanconi anemia pathway) as a partner of FANCD2 (ferroptosis negative regulator, up) in COAD (Figure 5A). Reports suggest that the Fanconi anemia pathway plays a role in interstrand crosslink repair of cancer tissues [42]. The second target, NCOA4 in COAD (ferroptosis, up), interacts with RXRA (apoptosis). It is shown that RXRA induces cell death in tumor tissues [43]. In GBM, we found that KEAP1 (apoptosis) interacts with IKBKB (alkaliptosis, down) (Figure 5B). It is reported that KEAP1 regulates cancer cell growth [44]. We observed the ARHGEF2 (RHOA signaling) connected with RHOA (entotic cell death, down) in GBM and SCLC (Figure 5C). ARHGEF2 plays a role in cell migration in tumor tissues [45]. To identify the small molecules to target the PPI, we need to understand the PPI binding site using protein–protein docking. Thus, we predicted the binding site responsible for each drug target and its interacting partner in COAD, GBM, and SCLC (Figure S9A–C).

Our study aims to identify the reliable drug molecules that could be used directly for experimental validation in the future. In brief, we used following parameters: first we extracted the drugs from the CheMBL database for each drug target based on the Lipinski rule of 5 (RO5), which deals with drug likeliness of molecules [46]. Second, we considered drug molecules with anti-cancer potential for further analysis. Here, we show that Doxorubicin hydrochloride (D-HCL) targets FANCD2 in COAD, and interestingly, Doxorubicin induces cell death in cancer cells [47]. This reveals that D-HCL has the potential to attenuate cancer growth. Lenvatinib targets NCOA4 in COAD; this drug is known to reduce tumor growth, and it is tested in phase 2 clinical trials to treat gastric cancer patients [48,49]. In GBM, IKBKB can be targeted by wortmannin. This molecule curbs cancer cell proliferation and promotes cell death [50]. Clausine E binds to RHOA in GBM and SCLC. Interestingly, this small molecule belongs to the class of clausines which are known to impart anti-proliferative potential in cancer cells [51]. This study provides a view of non-apoptotic cell death interactions and predicted drug targets intertwined with proteins involved in tumor growth pathways. Our analysis also revealed small molecules that could bind to the drug targets, and it paves a path to experimental validation to modulate the protein–protein interactions of NACDGs in cancer cells.

## 4. Materials and Methods 

### 4.1. RNA-Sequencing Data Analysis

We retrieved RSEM normalized expression data related to COAD, GBM normal, and cancer patients from The TCGA firehouse (https://gdac.broadinstitute.org/). The SCLC patient gene expression dataset (normal and cancer) acquired from the GEO database, deposited under accession number: GSE60052 [21], raw reads were further processed by the GRIEN database [52]. All the data analysis was performed separately for each cancer. We performed principal component analysis using the ‘prcomp’ R function. DEGs identified using normalized expression data through the DESeq2 package, which is a part of integrated Differential Expression and Pathway analysis (iDEP v.90) software [53]. We considered genes as a DEG that satisfied the following criteria: log_2_ fold change 1 (absolute fold change: 2) and *p*-adjusted-value ≤ 0.05.

### 4.2. Literature Mining of Non-Apoptotic Cell Death Genes

We performed literature mining [54] to extract NACDGs related to following pathways: necroptosis, pyroptosis, ferroptosis, parthanatos, entotic cell death, netotic cell death, lysosome dependent cell death, autophagy-dependent cell death, alkaliptosis, and oxeiptosis. We pulled 71 NACDGs from table 1 [54]. We overlapped these NACDGs with DEGs extracted from COAD, GBM, and SCLC and focused on this subset of non-apoptotic cell death genes for further analysis.

### 4.3. Transcription Factor Network Analysis

Transcription factor (TF) enrichment analysis was done using TRRUSTv2 (https://www.grnpedia.org/trrust/) and Chea3 tool (https://maayanlab.cloud/chea3/) [22,23]. Initially, we logged NACDGs on to TRRUST v2 to retrieve the TFs connected to NACDGs. If we did not find a NACDG in TRRUST, we uploaded them to the Chea3 tool to extract the TFs. Finally, we merged the TF-targets networks downloaded from TRRUSTv2 and Chea3 tool, and they visualized them as networks using Cytoscape v3.7.1 [55]. We computed each TF’s degree to identify the key TF in each network using network analyzer cytoscape plugin.

### 4.4. Protein–Protein Interaction Network Analysis

We used three high-quality resources to extract protein–protein interactions of differentially expressed NACDGs in COAD, GBM, and SCLC by uploading them to the following databases: first, we uploaded NACDGs to HuRI (Human Reference Interactome map), which contains ~50,000 interactions (http://www.interactome-atlas.org) [56]. If protein interaction of NACDG was not found in HuRI, then we connected them to the second database, HINT, which contains ~53,000 high quality physical binary protein–protein interactions (http://hint.yulab.org) [57]. Finally, if we did not find protein interaction of NACDG in the HuRI and HINT databases, we used the STRINGV11 database to extract protein interaction data for those NACDGs (https://string-db.org) [58]. This database consists of all known and predicted protein–protein interactions based on the experimental, text mining and co-expression evidence of human organism. We retrieved the interactions with a 0.7 confidence score from STRING DB. The PPI networks were pictured in Cytoscape v3.7.1 [55]. 

### 4.5. Drug Target Prediction from Protein–Protein Interaction Networks and Validation

We used the graph theory to analyze the topological importance of a node in protein–protein interaction networks. This approach can assess the vitalness of each protein in the network. In our study, we utilized the four topological metrics to predict drug targets. We used the following parameters to identify the hub proteins: we computed degree by counting the number of links of each node (protein). Kleinberg’s hub score is based on the following notion where a graph k and hubs of k identified by adjacency matrix b of k by calculating the eigenvectors [59]. In order to validate hubs identified by Kleinberg’s hub score and degree, we calculated two centrality parameters closeness and betweenness. The closeness used to assess the overall vicinity of a node (protein) to other nodes in the PPIN in turn measures the effectiveness of the communication of the node [60]. Betweenness computes the nonredundant shortest paths passing through a node. Elevated betweenness indicates the central proteins in the PPIN [61]. To mine drug targets from these hub proteins, we further analyzed them with eccentricity and coreness metrics [18]. We validated drug target proteins as follows: we uploaded these target proteins to databases such as Drug Bank (https://go.drugbank.com) [62], TTD (http://db.idrblab.net/ttd/) [63], DINIES (https://www.genome.jp/tools/dinies/) [63], and Cancer DR (http://crdd.osdd.net/raghava/cancerdr/) [64]. Drug target not found in none or found in one of the databases was considered as a new target for further analysis.

### 4.6. Identification of miRNA Regulating Drug Targets

To extract the cancer-specific (Colon adenocarcinoma, Glioblastoma multiforme, and Small Cell Lung Cancer) miRNAs, we used dbDEMC v2.0 (https://www.picb.ac.cn/dbDEMC/) [65]. dbDEMC also provides log_2_ fold changes for each miRNA derived from gene expression studies. We culled miRNAs (Human organism) with drug targets proteins obtained from PPIN networks characteristic to each cancer from miRWalk v3.0 (http://mirwalk.umm.uni-heidelberg.de) [66]. We merged miRNAs from dbDEMC with miRNA-drug target networks. Finally, the most upregulated miRNAs are retained for further analysis.

### 4.7. Pathway Enrichment Analysis of Networks

We performed the pathway analysis of proteins from interaction networks using METASCAPE (https://metascape.org/gp/index.html#/main/step1) [67] which contained the KEGG pathways, gene ontology biological process. The pathways considered statistically significant using a *p*-adjusted-value ≤ 0.05.

### 4.8. Drug Target Interaction Network Analysis and Prediction of PPI Binding Sites Identification of Drugs

We derived the protein interacting partners of drug target proteins from protein–protein interaction networks. We extracted the direct interacting partners of each drug target responsible for tumor-related pathways. Experimental based structures of drug targets (COAD: FANCD2-FANCI complex, PDB id- 6VAD, GBM: IKBKB, PDB id- 4KIK, GBM and SCLC: RHOA, PDB id- 1CC0) and their interacting partners (COAD: RXRA, PDB id- 6JNO, GBM: KEAP1, PDB id- 6SP1, GBM and SCLC ARHGEF2, PDB id- 5EFX) and their interacting partners were downloaded from PDB database (https://www.rcsb.org/). We removed the ligands present in these .PDB files and took only structures for analysis. To model NCOA4 3D structure a drug target in COAD, we used the following procedure: first retrieved the sequence of NCOA4 in fasta format from the Uniprot database (https://www.uniprot.org) [68] (uniport id: Q13772, homo sapiens). This sequence was given as an input to the online ITASSER software (https://zhanglab.ccmb.med.umich.edu/I-TASSER/) [69] to model the 3D structure NCOA4. The model aligned with PDB id: 6N7PX (S. cerevisiae spliceosomal E complex, aligned residues coverage score: 0.94) NCOA4 model was retrieved and minimized using the ModRefiner tool (https://zhanglab.ccmb.med.umich.edu/ModRefiner/) and corrected side chains of the structure via Chiron tools (https://dokhlab.med.psu.edu/chiron/login.php) [70,71]. We used ProSA-web (https://prosa.services.came.sbg.ac.at/prosa.php) [72] to compute the z-score of the NCOA4 structure to compare it with experimental derived structures (X-ray or NMR). To identify the binding site residues between the drug target and interacting partner, we performed protein–protein docking using HDOCK online server [73]. We extracted drugs from the CheMBL online database (https://www.ebi.ac.uk/chembl/) [74] based on the Lipinski rule of 5 (RO5), which measures drug likeliness of small molecules [46] and known cancer drugs. Finally, drug molecules that satisfied these parameters and target the drug target proteins from cancer datasets were retained for further analysis.

### 4.9. Statistical Analysis and Data Visualization

The statistical analysis and visualization in this study were done using the R statistical software v3.5.1 (https://www.r-project.org/). The principal component analysis was visualized as a scatter plot using the ggplot2 R package. We computed z-score of expression for NACDGs in each cancer using heatmap.2 function in gplots R package. Common and unique genes/proteins were visualized as a barplot using the UpSet R package. We plotted the node degree distribution of protein–protein interaction networks using the igraphv1.0.0 R package (https://igraph.org/r/). Statistical evaluation of power-law distributions done using Clauset’s method [75]. This method uses a goodness-of-fit test based on the Kolmogorov–Smirnov (KS) statistic to compute the null hypothesis. This hypothesis rejected with significance (*p*-value < 0.1), which done with power law fit function in the igraph package in R. We used igraph R package to compute the Kleinberg’s hub score, degree, closeness, betweenness, eccentricity, and coreness. Scatter plots related to hub analysis and barplots related to the number of NACDGs and pathway enrichment analysis, were rendered using the ggplot R package. Heatmaps generated using the pheatmap R package. We utilized Pymol v2.7 software to visualize the protein–protein complexes.

## 5. Conclusions

This study took advantage of publicly available apoptosis-resistant colon adenocarcinoma (COAD), glioblastoma multiforme (GBM) and small cell lung cancer (SCLC), transcriptome datasets, and performed gene expression and layered network analyses. This approach highlighted non-apoptotic cell death genes as well as vital transcription factors and miRNA regulating these genes. Through the protein–protein interaction network analysis, we connected non-apoptotic cell death proteins with various tumorigenic functions and predicted drug targets along with small molecules. Our study lays the foundation for more comprehensive experimental study (in vitro, in vivo) on modulating the cell death drug targets FANCD2, NCOA4 (COAD), IKBKB, (GBM), and RHOA (GBM and SCLC), to improve the therapeutic avenues in cancer treatment.

## Supplementary Materials

The following are available online at https://www.mdpi.com/2072-6694/13/4/851/s1, Figure S1: Protein–protein interaction networks regulated by cell death genes in COAD, GBM and SCLC cells. Figure S2: Topological analysis of cell death regulated protein–protein interaction networks (PPIN) in the COAD, GBM and SCLC. Figure S3: Pathway enrichment analysis of PPINs. Figure S4: Hub validation analysis using closeness and betweenness. Figure S5: Pathway enrichment analysis of drug target networks. Figure S6: Drug target database validation analysis. Figure S7: miRNA and drug target networks. Figure S8: Structure model of NCOA4 protein. Figure S9: Drug target and interacting partner docking analysis. Table S1: Significantly up and down regulated genes in COAD. Table S2: Significantly up and down regulated genes in GBM. Table S3: Significantly up and down regulated genes in SCLC. Table S4: list of NACDGs in cancer datasets. Table S5: Common and unique NACDGs across the cancer datasets. Table S6: Common and unique hubs across the cancer datasets.
