# Supplementary material for: Network Analysis Identifies Drug Targets and Small Molecules to Modulate Apoptosis Resistant Cancers
Source: Cancers (Basel). 2021 Feb 18;13(4):851. doi: 10.3390/cancers13040851 (PMC7922238; doi:10.3390/cancers13040851)
Supplement: Supplementary file 1 [file cancers-13-00851-s001.zip › SupplementaryFigures_tables/Supplementaryfigures.pdf]

Supplementary Figure 1

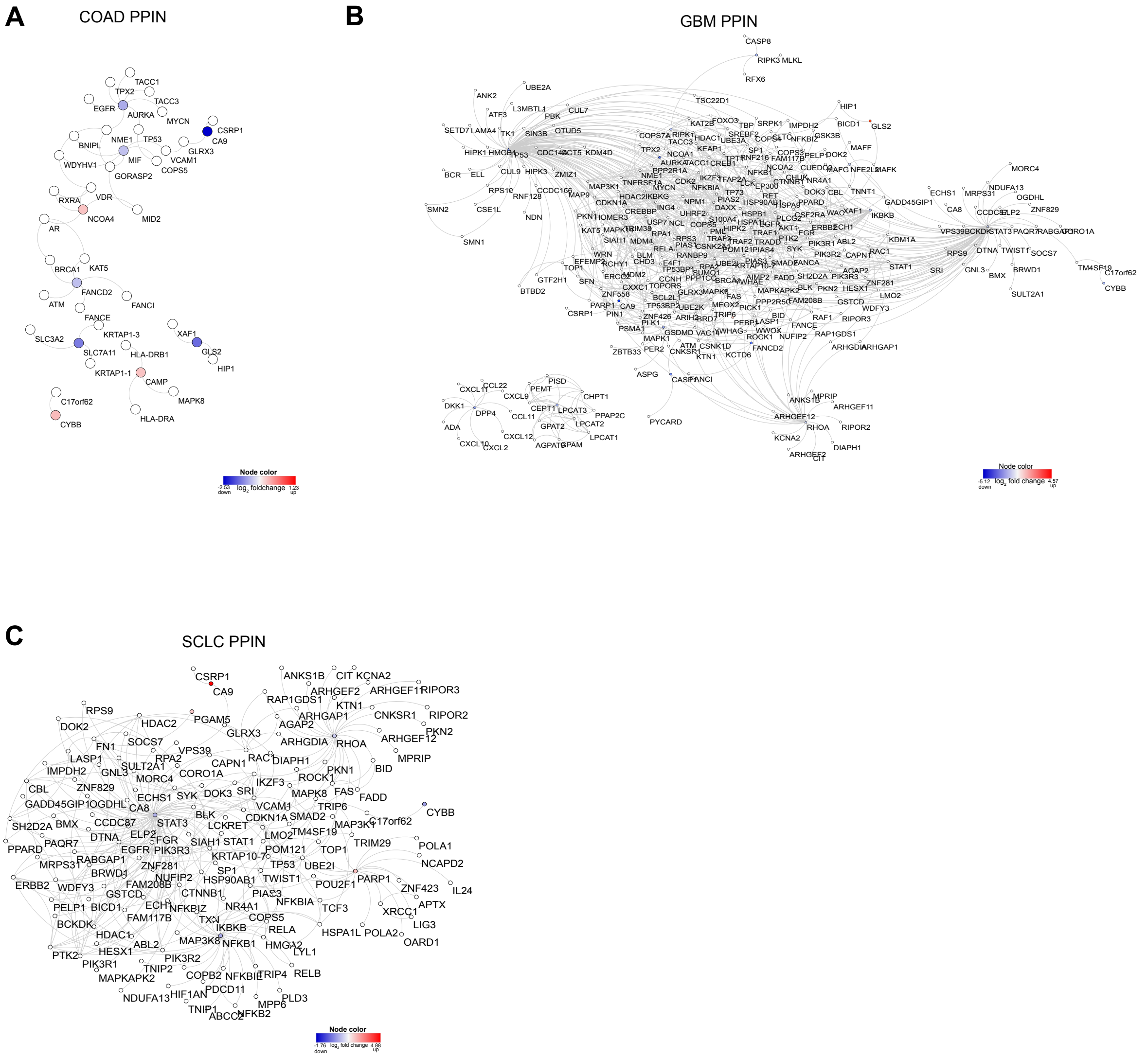

Figure S1: Protein-Protein interaction networks regulated by cell death genes in COAD, GBM, and SCLC cells. A-C) Protein-protein interaction network of cell death differentially regulated genes in cancers visualized using the Cytoscape v3.7.1. The circle represents a protein, and the grey edge connecting two proteins specifies the interaction between the proteins. Note: Red and blue color denote up and down-regulation.

Supplementary Figure S2

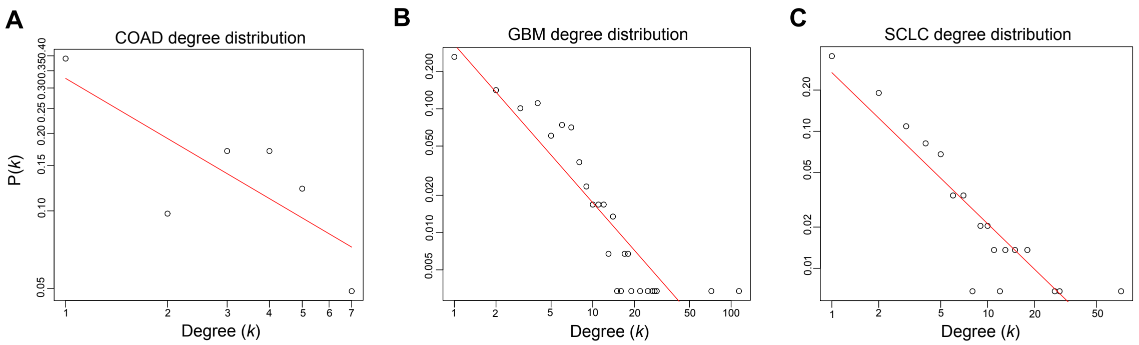

Figure S2: Topological analysis of cell death regulated protein-protein interaction networks (PPIN) in the COAD, GBM, and SCLC. A-C) Scatter plots represent the node degree distribution plotted against the number of nodes in each cancer network. P(k) is the number of proteins with k links.

Supplementary Figure S3

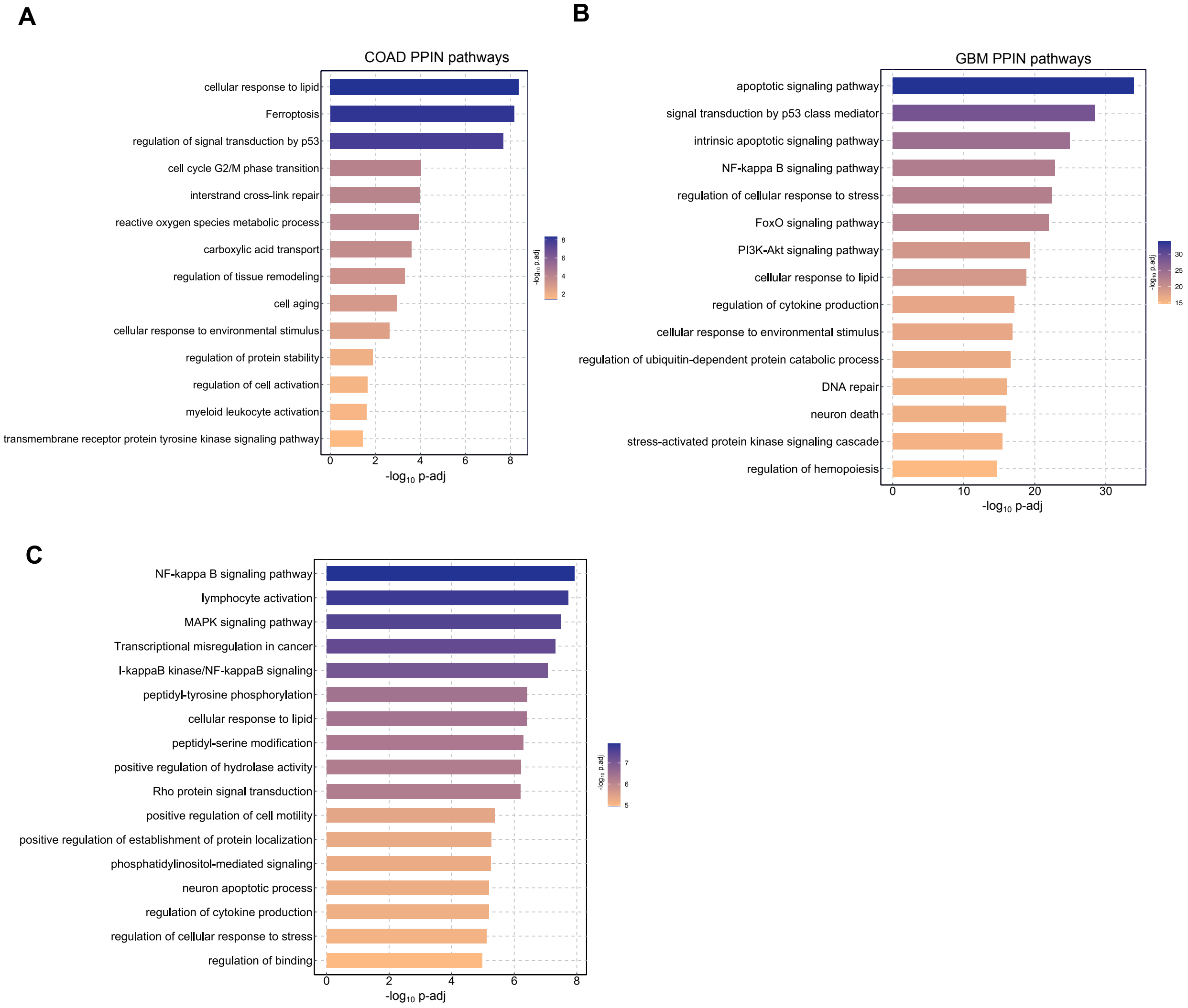

Figure S3: Pathway enrichment analysis of PPINs. A-C) The bar plots represent the most significant (P≤ 0.05) pathways in COAD, GBM, and SCLC cell death regulated PPIN.

Supplementary Figure S4

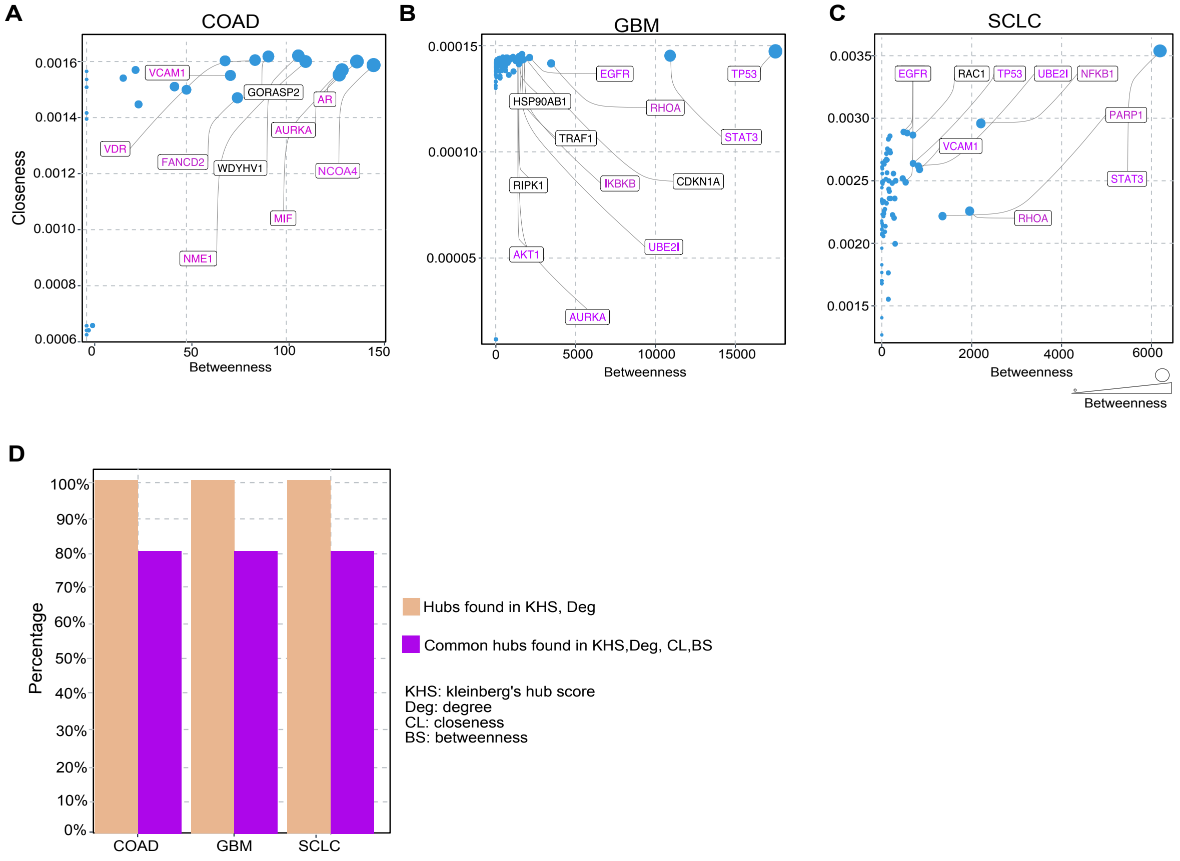

Figure S4: Hub protein validation analysis of NACDP protein-protein interaction networks. (**A-C**) The centrality analysis of COAD, GBM, and SCLC protein-protein interaction networks, including closeness and betweenness. Note: Purple color shows the common proteins with the top 10 proteins found in degree and Kleinberg hub score. (**D**) Percentage of common hub proteins between the top 10 proteins found in degree and Kleinberg hub score and closeness and betweenness.

Supplementary Figure S5

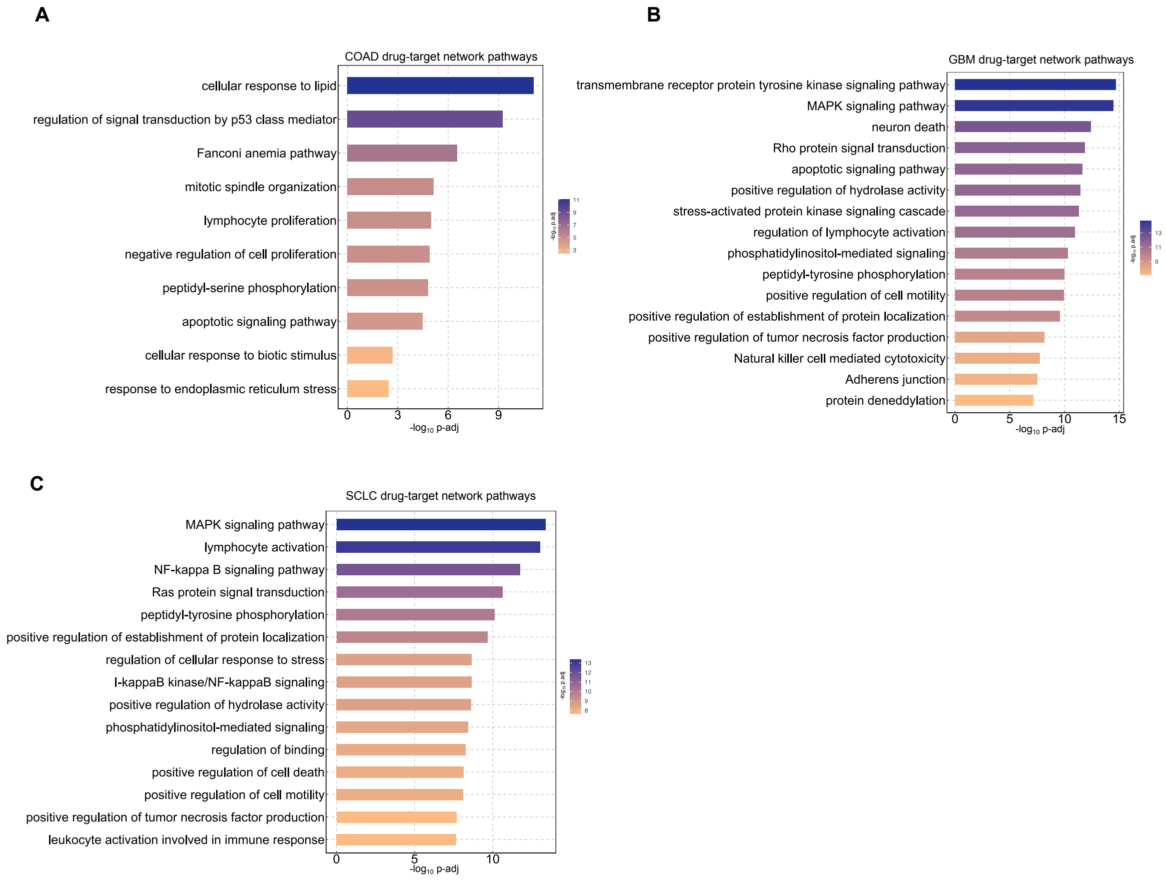

Figure S5: Pathway enrichment analysis of drug-target networks. A-C) The significant ($P \leq 0.05$) pathways in COAD, GBM, and SCLC cell death drug-target networks.

Supplementary Figure S6

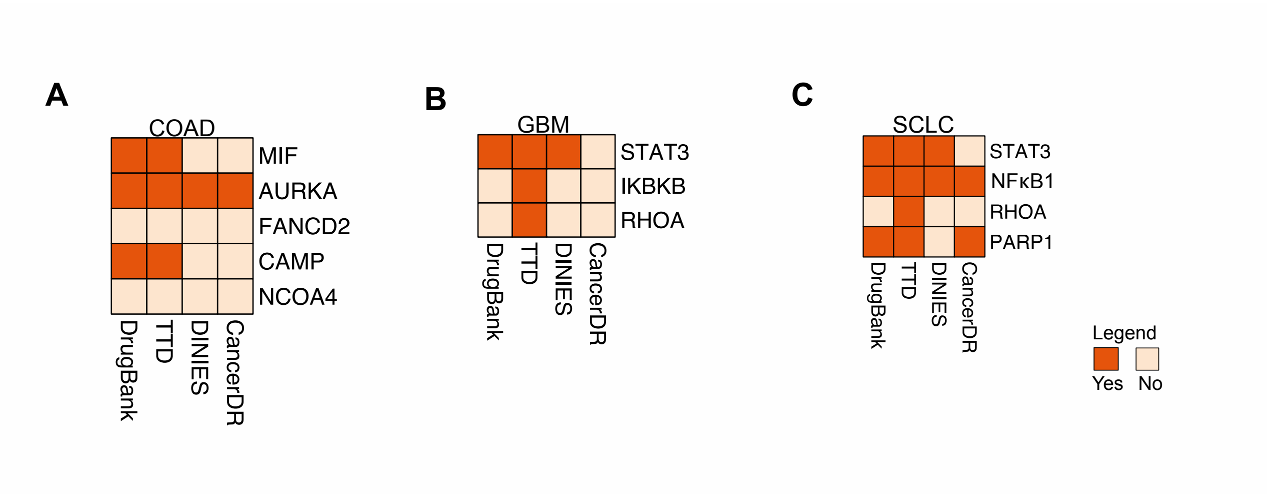

Figure S6: Drug target database validation analysis. A-C) These heatmaps represent the drug target database analysis of predicted drug targets in COAD, GBM, and SCLC. Note: Red and blue color denote up and down-regulation.

Supplementary Figure S7

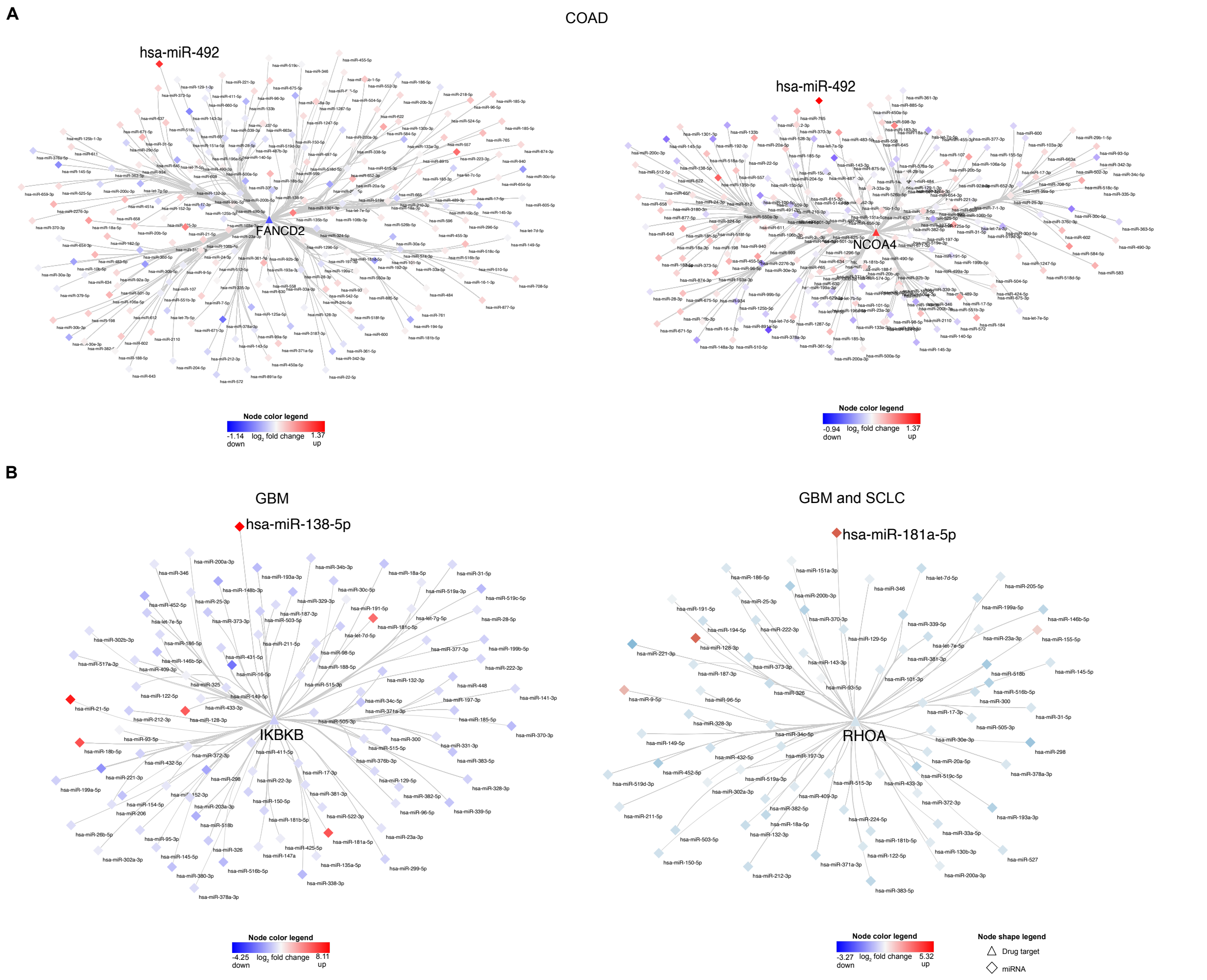

Figure S7. miRNA-drug target networks. (**A-B**) These networks denote the miRNA interactions with drug targets in COAD, GBM, and SCLC. Note: node color red and blue symbolize the up and down-regulation.

Supplementary Figure S8

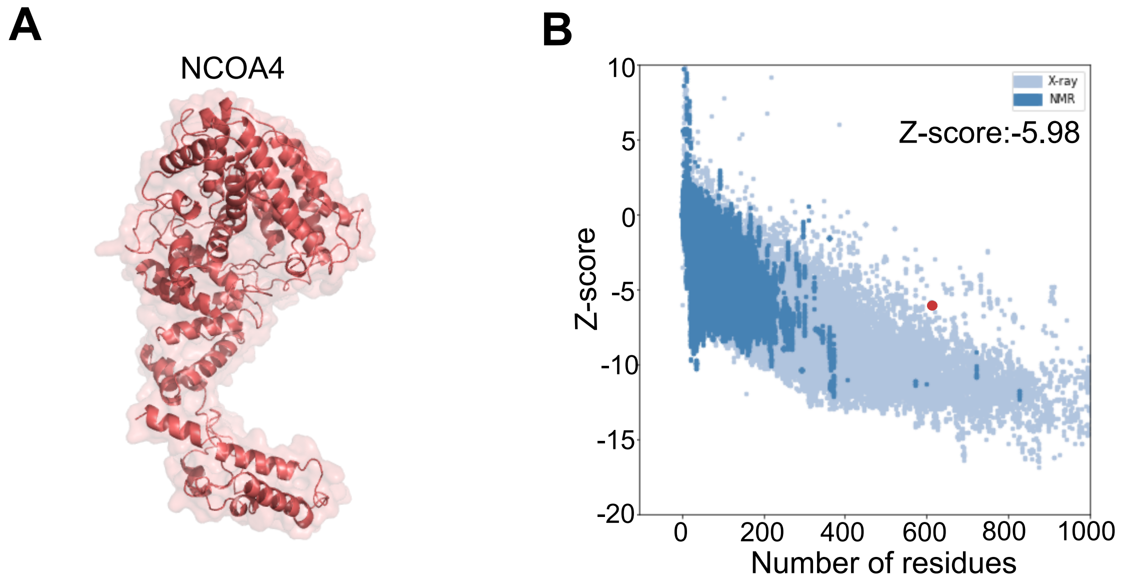

Figure S8: Structural model of NCOA4 and validation. A) The 3D model of NCOA4 using ITASSER. B) 3D models structure corroboration by ProSA. This plot represents modeled structure score, NCOA4 specified by the chocolate dot, revealed that it is in the range of scores found on analogous sized proteins with an X-ray quality.

Supplementary Figure S9

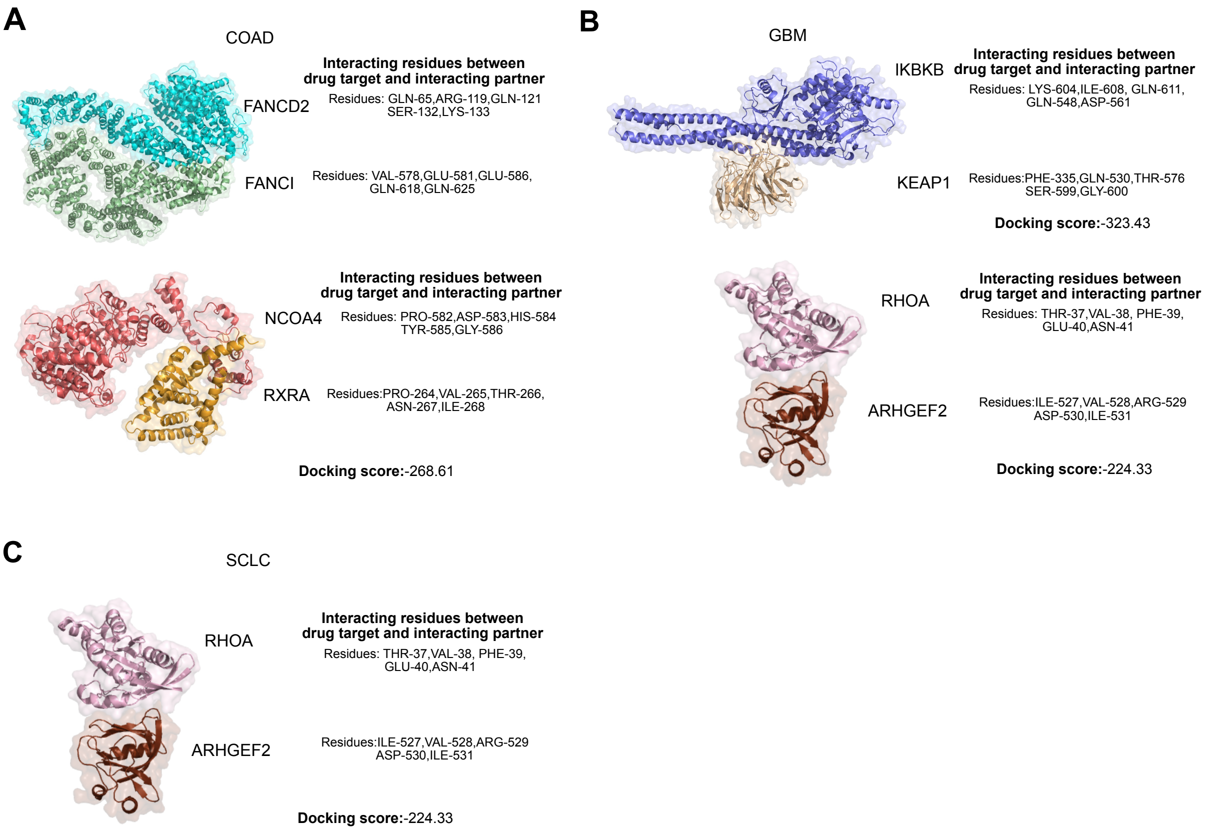

Figure S9: Drug target and interacting partner docking analysis. A-C) The structures represent interaction analysis between the drug target and its interacting partner in COAD, GBM, and SCLC. Docking scores computed using HDOCK.
